# Quality of Life Among Informal Caregivers of Patients With Degenerative Cervical Myelopathy: Cross-Sectional Questionnaire Study

**DOI:** 10.2196/12381

**Published:** 2019-11-07

**Authors:** Oliver Daniel Mowforth, Benjamin Marshall Davies, Mark Reinhard Kotter

**Affiliations:** 1 Division of Neurosurgery Department of Clinical Neurosciences University of Cambridge Cambridge United Kingdom

**Keywords:** spinal cord diseases, spondylosis, spinal osteophytosis, surveys and questionnaires, quality of life, chronic disease

## Abstract

**Background:**

Degenerative cervical myelopathy (DCM) is a common, chronic neurological condition that severely affects individuals by causing a range of disabling symptoms, frequently at a time around the peak of their careers. Subsequently, individuals with DCM often become dependent on informal care arrangements. The significant economic contribution of informal care and its burden on care providers are becoming increasingly recognized.

**Objective:**

This study aimed to measure the quality of life of DCM informal caregivers and provide preliminary insight into possible contributing factors.

**Methods:**

Carers of individuals with DCM completed a Web-based survey hosted by Myelopathy.org, an international DCM charity. Carer quality of life was assessed in the form of caregiver happiness and 7 dimensions of carer burden using the Care-Related Quality of Life (CarerQol) instrument. The relationships between patient disease severity, patient pain, and carer quality of life were investigated. Differences in carer quality of life were assessed across patient and carer demographic groups, including between UK and US carers.

**Results:**

DCM caregivers experienced substantial burden as a result of their caregiving (mean CarerQol-7D=64.1; 95% CI 58.8-69.5) and low happiness (mean CarerQol-VAS [Visual Analog Scale]=6.3; 95% CI 5.7-6.9). Burden was high and happiness was low in DCM carers when compared with a large, mixed-disease study of adult informal carers where CarerQol-7D was 79.1 and CarerQol-VAS was 7.1. No significant relationship was found between DCM carer quality of life and patient disease severity and pain scores. DCM carer quality of life appeared uniform across all patient and carer demographic groups.

**Conclusions:**

Caring for individuals with DCM is associated with reduced quality of life in the form of significant burden and reduced happiness. Reductions appear greater in DCM than in other diseases investigated. However, no simple relationship was identified between individual patient or carer factors and carer quality of life.

## Introduction

### Background

Degenerative cervical myelopathy (DCM) is a neurological condition of symptomatic cervical spinal cord compression, secondary to a range of degenerative changes in the cervical spine [1]. The causative pathology includes osteophyte formation, disc herniation and ligament hypertrophy, calcification, and ossification [2].

DCM is the most common spinal cord disorder [2], with evidence from imaging studies estimating prevalence as high as 5% in the over 40s [3]. At present, treatment is limited to surgical decompression, which is able to halt disease progression, but existing damage is often permanent [4,5]. Consequently, most patients retain lifelong disabilities, including reduced quality of life. In fact, a recent study demonstrated that DCM patients have one of the most impaired quality of life scores of all chronic diseases—lower than diabetes, cancer, chronic obstructive pulmonary disease (COPD), and depression according to 36-Item Short Form Heath Survey (SF-36) scores [6].

Informal care refers to care provided by family and friends [7]. Historically, informal care received little attention. However, it is clear that providing informal care brings substantial burdens to informal care providers. One study found a 63% higher mortality risk among carers experiencing strain compared with noncaregiving controls [8], whereas another study reported a significant association between carer depression and the likelihood and amount of time missed at work [9]. Informal carers of patients suffering from a range of chronic conditions, including spinal cord injuries [10], dementia [11], eating disorders [12], and Pompe disease [13], among others, have now been studied. Regardless of the disease, these studies consistently describe increased levels of burden, stress, depression, physical health problems, and a range of psychosocial problems in carers. Moreover, in a national survey of 2000 carers in the United Kingdom, 77% reported health deterioration because of the strain of providing care [14]. Unfortunately, despite these substantial burdens for carers, informal care has a minor influence on health care decisions [7].

Despite this, recent work continues to demonstrate that informal care frequently forms a significant proportion of total care, particularly in chronic conditions [7] such as DCM. For example, in the United Kingdom there are an estimated 6 million informal carers [15], and in the United States, estimates suggest that there may be as many as 66 million informal carers [16]. The total economic contribution of UK carers is estimated at £132 billion per year [17], whereas in the United States the total opportunity costs of informal elder-care amounts to US $522 billion annually [18].

Methods have been developed to measure informal care and facilitate its incorporation into economic evaluations of health care [19]. Available methods are diverse, with a range of different instruments, measurements, and valuation techniques available. Both costs and effects of informal care can be measured with a range of monetary and nonmonetary methods. Nonmonetary methods focus on areas such as health-related quality of life, care-related quality of life, and well-being. Monetary methods include revealed preference-based methods and stated preference methods.

**Figure 1:**
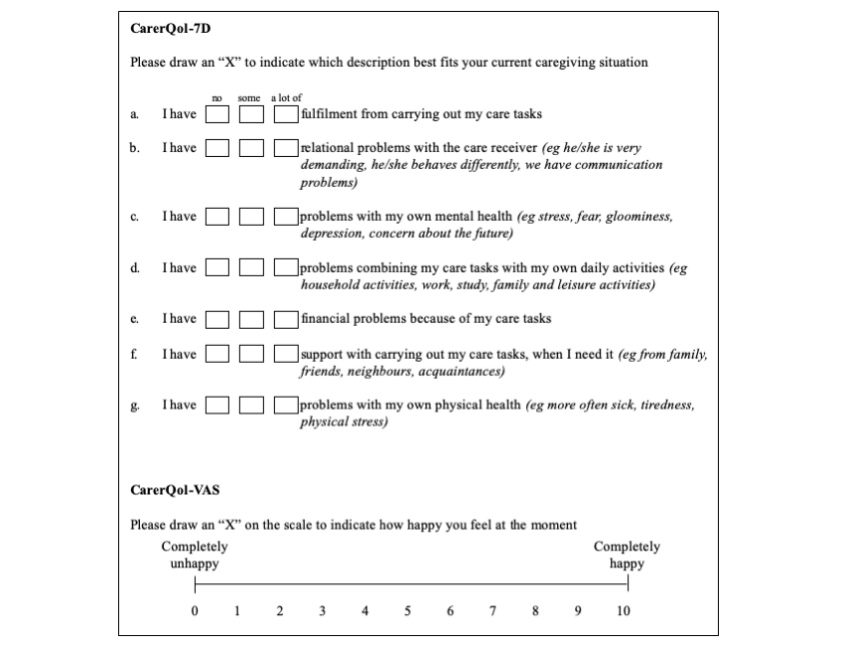
The Care-Related Quality of Life (CarerQoL-7D) instrument assesses carer quality of life in terms of burden and happiness (Visual Analog Scale [VAS]). For the 7D, carers choose from 3 responses to 7 different questions, their overall score aggregating to a total out of 100, with 100 representing the least carer burden. For the VAS, carers rate their happiness on a visual analog scale from 0 to 10, with 10 representing the greatest happiness.

The Care-Related Quality of Life (CarerQol) instrument was designed to measure and value the impact of providing informal care [20]. The instrument consists of 2 parts. The first part, CarerQol-7D, is a subjective burden measure assessing 2 positive and 5 negative dimensions of the impact of providing care (Figure 1). The 2 positive dimensions measure (1) fulfillment from caregiving and (2) support with caregiving. The 5 negative dimensions assess (1) relationship problems, (2) own mental health problems, (3) problems combining care tasks with daily activities, (4) financial problems, and (5) own physical health problems.

The second part, CarerQol-Visual Analog Scale (VAS), consists of a subjective measure of carer well-being in the form of a general happiness rating on a VAS of 1 to 10. A particular benefit of the CarerQol is that it is generic, allowing for comparison between conditions.

### Objectives

Given that DCM causes substantial reductions in the quality of life of patients [6], we hypothesized that there would also be reductions in the quality of life of DCM carers.

The objective was to measure the quality of life of DCM informal carers and provide preliminary insight into possible contributing factors.

## Methods

The DCM carer quality of life survey was designed and is reported following the Checklist for Reporting Results of Internet E-Surveys [21].

### Survey Design

A cross-sectional observational study was conducted utilizing a Web-based survey targeted at carers of patients with DCM.

The questionnaire included questions to assess the situation of DCM patients and carers. Patient situation questions captured demographics including age, gender, and education; Nurick score (a disease severity classification that utilizes the degree of walking impairment to score the severity of a patient’s cervical myelopathy) [22]; best, worst, and current neck and arm/hand pain scores; and patient dependence on a carer. Carer situation questions captured carer demographics including age, gender, education, employment, country of residence, and length of time as a carer.

Carer quality of life was assessed using the CarerQol instrument [20]. The instrument (Figure 1) comprises 2 parts: a CarerQol-7D and a CarerQol-VAS.

The CarerQol-7D is designed to provide a comprehensive description of the caregiving situation [23]. Each dimension is assessed using a 3-item scale. For example, the respondent rates the statement “I have fulfillment with carrying out my care tasks” by choosing from 3 responses: (1) no, (2) some, or (3) a lot of. The responses for each dimension are weighted with a tariff score. From these, an aggregate score between 0 and 100 can be calculated, with 0 corresponding to the most carer burden (worst informal care situation) and 100 to the least carer burden (best informal care situation). Tariffs for calculating these utility scores were derived using discrete choice experiments among the general population and are population-specific. At the time of study, tariffs were available for the Netherlands [24], Australia, Germany, Sweden, the United Kingdom, and the United States [25].

The CarerQol-VAS consists of a horizontal visual analog scale whereby the carer rates their happiness on a scale of 0 to 10, with 0 representing a situation in which the carer is completely unhappy and 10 representing a situation in which the carer is completely happy [20]. The CarerQol-VAS allows informal care to be considered from an economic perspective. Carer happiness assessed using the CarerQol-VAS is a broad measure, which allows capturing of the influences of the multitude of factors that contribute to carer happiness.

Numerous construct validation studies support the validity of the CarerQol instrument [26-29].

The survey was initially piloted on a small group of carers and found to be satisfactory without need for modification.

### Ethics Approval and Informed Consent

The study was ethically approved by the University of Cambridge. All research was performed in accordance with relevant guidelines and regulations.

All carers completed the questionnaire voluntarily and were informed before doing so that their responses would be used anonymously for research purposes. Study objectives were outlined on the initial page, including details of the host organization. This acted as the electronic consent, with continuation into the survey taken as agreement. Respondents were also presented with a description of DCM, including relevant symptoms, and were required to confirm they cared for someone who suffered from this condition

No respondent-identifiable information was stored.

### Participants

All DCM carers from countries for which CarerQol tariffs were available at the time of study were included. Carers from countries without available CarerQol tariffs were excluded.

### Recruitment

The recruitment strategy has been described previously [30]. An open survey design was employed. Carers of patients with DCM were recruited to an Web-based questionnaire, administered by SurveyMonkey. Social media posts (Facebook and Twitter), supported by Myelopathy.org, were utilized to recruit participants. No contact was made with participants outside the survey.

### Administration

The questionnaire was hosted on a designated landing page on Myelopathy.org, a UK registered charity, with a large online, international patient community. The website provides a range of educational materials, support groups, and details of current research studies. The survey was not administered via email. Completion of the survey was voluntary, and no incentives were offered. Responses were collected for 12 months from November 1, 2015. A total of 25 survey items were distributed over 7 survey pages. Only responses that were complete for all carer quality of life domains were included in the final data analysis. A missing data analysis showed complete and incomplete responses were otherwise comparable, providing reassurance that excluding incomplete responses did not introduce additional bias. Respondents were able to click back though and review their answers to previous questions before submission of the survey.

### Response Rates

Google Analytics, a Web-based analytics service that enables tracking of visits to a website, was utilized in this study to measure the number of visitors to Myelopathy.org. Survey view rate for total visits to the Myelopathy.org home page was 2.37% (421/17,737), participation rate was 32.3% (136/421), and completion rate was 37.0% (136/368).

### Preventing Multiple Entries From the Same Individual

Internet protocol addresses were recorded and used to prevent users submitting multiple responses.

### Statistical Methods

Data were checked for the presence of outliers and normality of distribution. First, as CarerQol, Nurick, and pain scores were not monotonically related, Kendall tau-b was utilized to assess for a relationship, using 2-tailed tests. Second, differences in CarerQol-7D and VAS scores between demographic groups were assessed using 1-way analysis of variances (ANOVAs). In 1 case the assumption of homogeneity of variance was violated; a Welch ANOVA was performed to assess for difference in CarerQol-VAS as a function of duration of time as a carer. Third, an independent-samples *t* test was run to determine if there was a difference in CarerQol-7D score between UK and US carers. Fourth, as CarerQol-VAS violated the assumption of normality, a Mann-Whitney *U* test was run to determine if there were differences in CarerQol-VAS between UK and US carers. Distributions of UK and US CarerQol-VAS scores were similar, as assessed by visual inspection. Analyses were conducted using SPSS Version 22 (IBM Corp). Significance was set at *P*<.05. We report mean (SD) unless otherwise specified.

## Results

### Participants

**Figure 2:**
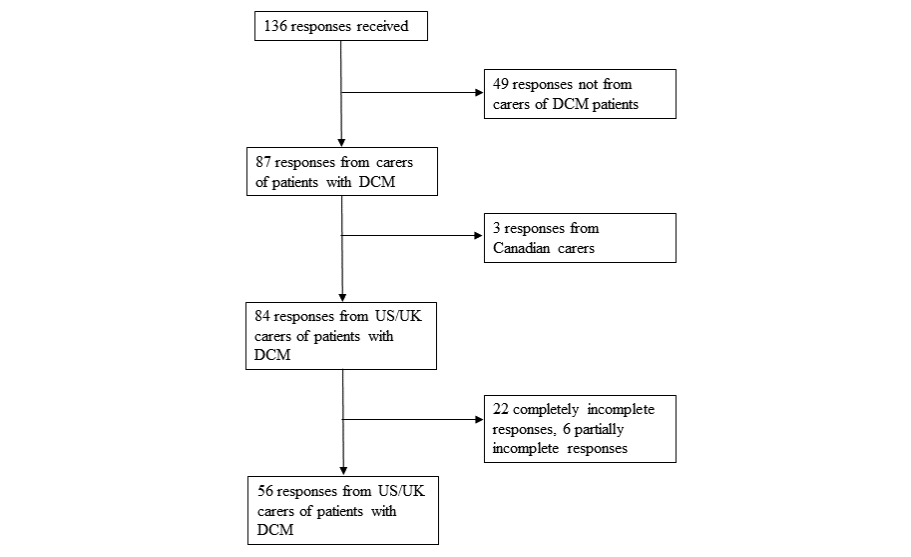
Flowchart of response selection. A total of 136 questionnaire responses were received, 56 of which were included in the final analysis. DCM: degenerative cervical myelopathy.

A total of 136 responses were received (Figure 2). A total of 49 responses were inadvertently completed by DCM patients and were thus excluded. Of the remaining 87 responses, 31 were excluded: 3 were completed by Canadian carers for whom CarerQol tariffs were not available at the time of study, and 28 responses had partial (6) or completely (22) missing CarerQol data. In a missing data analysis, scores for each CarerQol component between the 56 complete and 6 partially incomplete responses were comparable, providing reassurance that excluding the partially incomplete responses was unlikely to introduce bias. Patient and carer characteristics were >95% complete for each variable, permitting meaningful analysis.

### Patient and Carer Situation

As summarized in Table 1 (n [%]), the majority of carers were female (32/53, 60%), white (51/52, 98%), and from the United Kingdom (45/56, 80%). Duration of time as a carer was varied, ranging from less than 1 year to 25 years. Most (49/54, 91%) carers were over the age of 40 years, and most (31/53, 59%) combined their caring responsibilities with either full-time (21/53, 40%) or part-time (10/53, 19%) work. The majority (19/34, 56%) felt their employment had been affected by the demands of their caregiving.

**Table 1:** Patient and carer situation.

Variable		Patient, n (%)	Carer, n (%)
**Gender**		**n=56**	**n=53**
	Male	22 (39)	21 (40)
**Country of residence**		**n=56**	**n=56**
	United Kingdom	45 (80)	45 (80)
	United States	11 (20)	11 (20)
**Age (years)**		**n=56**	**n=54**
	≤17	0 (0)	2 (4)
	18-20	0 (0)	0 (0)
	21-29	1 (2)	0 (0)
	30-39	4 (7)	3 (5)
	40-49	14 (25)	13 (24)
	50-59	18 (32)	20 (37)
	≥60	19 (34)	16 (30)
**Education**		**n=56**	**n=53**
	Less than high school	14 (25)	6 (11)
	High school degree or equivalent	10 (18)	14 (26)
	Some college but no degree	15 (27)	14 (26)
	Associate degree	5 (9)	5 (10)
	Bachelor’s degree	10 (18)	4 (8)
	Graduate degree	2 (3)	10 (19)
**Ethnicity**		—^a^	**n=52**
	White	—	51 (98)
	African American	—	1 (2)
**Employment status**		—	**n=53**
	Employed, working full time	—	21 (40)
	Employed, working part time	—	10 (19)
	Not employed, looking for work	—	0 (0)
	Not employed, not looking for work	—	6 (11)
	Retired	—	14 (26)
	Disabled/unable to work	—	2 (4)
Employment affected by caring		—	19 (56)
**Length of time as carer (years)**		—	**n=56**
	0-1	—	13 (23)
	1-3	—	15 (27)
	3-10	—	24 (43)
	10-25	—	4 (7)
	>25	—	0 (0)

^a^Data were not collected.

### Carer Burden and Happiness

Mean carer burden measured by the CarerQol-7D was 64.1 (SD 20.4). Mean carer happiness assessed by the CarerQol-VAS was 6.3 (SD 2.2; Table 2).

**Figure 3:**
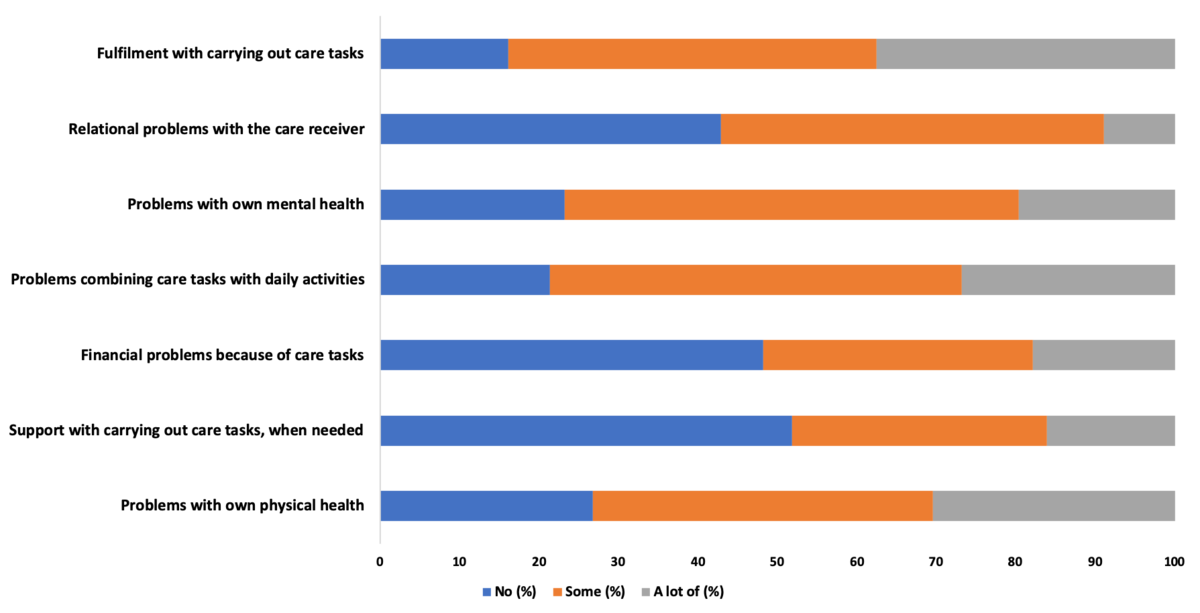
Distribution of responses to Carer-Related Quality of Life (CarerQol)-7D dimensions for degenerative cervical myelopathy carers.

The distribution of responses to individual CarerQol-7D dimensions, as a percentage of the overall responses to each dimension, is shown in Figure 3. Most DCM carers (47/56, 84%) experienced at least some (some or a lot of) fulfillment caring. Despite this, most carers experienced at least some mental health problems (43/56, 77%) and at least some physical health problems (41/56, 73%). In addition, around half reported at least some relationship problems with the care receiver (32/56, 57%) and at least some financial problems (28/56, 50%) because of their care responsibilities. Less than half (27/56, 48%) received at least some support with their care tasks, when needed. Most carers (44/56, 79%) experienced problems combining their care tasks with their everyday activities.

**Table 2:** Carer burden and happiness scores (n=56).

Instrument	Mean (SD)	95% CI	Range
Care-Related Quality of Life-7D	64.1 (20.4)	58.8-69.5	14.6-100
Care-Related Quality of Life-Visual Analog Scale	6.3 (2.2)	5.7-6.9	1-10

### Influence of Carer and Patient Situation

No significant relationship was found between carer burden or happiness and patient disease severity (Nurick score) and pain scores (Multimedia Appendix 1). Moreover, no significant difference in carer burden or happiness was found between different groups for demographic characteristics studied: patient age, gender, education, dependency, carer age, gender, education, employment, and duration of time as a carer (Multimedia Appendix 2).

### United States Versus United Kingdom

Mean DCM severity was similar for UK and US patients with Nurick scores of 2.93 (SD 1.03) and 2.91 (SD 0.94), respectively. Mean carer burden score was 58.2 in the United States compared with 65.6 in the United Kingdom (Table 3). Mean carer happiness score was 5.9 in the United States and 6.4 in the United Kingdom. These differences were not statistically significant.

**Table 3:** Mean carer burden and happiness lower in US carers.

Country and instrument		Mean (SD)	95% CI	Range
**United Kingdom (n=45)**				
	Care-Related Quality of Life-7D	65.6 (19.2)	60.5-70.6	22-100
	Care-Related Quality of Life-VAS	6.4 (2.3)	5.8-7.0	1-10
**United States (n=11)**				
	Care-Related Quality of Life-7D	58.2 (24.9)	51.7-64.7	14.6-95.5
	Care-Related Quality of Life-VAS	5.9 (1.9)	5.4-6.4	3-9

## Discussion

### Key Findings

These data demonstrate that carer quality of life is markedly reduced in DCM. Most carers reported mental and physical health difficulties, adverse impacts on employment, and strained relationships with the patient. No significant relationships between carer quality of life and patient disease and pain scores were identified. Moreover, no significant difference in carer quality of life was identified between different patient and carer demographic groups.

### Interpretation

These findings are significant because informal care plays a fundamentally important role in health care. In the United Kingdom, the value of informal care is estimated to be £132 billion per year [17], whereas according to the Institute for Fiscal Studies, in 2015/16 the UK government spent £140.6 billion on health and £29.9 billion on social care.

Increased strain on carers leads to increased carer illness, creating further demands on health care and negative economic consequences. As an age-related degenerative condition, the incidence and prevalence of DCM is expected to rise. In the context of an ageing population and accelerating health care innovations, health care costs are ever increasing. Therefore, supporting the providers of informal care, to prevent carer burnout and preserve carer health, is essential in ensuring sustainable and financially viable health care systems.

### Carer Quality of Life Is Reduced in Degenerative Cervical Myelopathy Compared With Other Chronic Conditions

Considering DCM carer quality of life in context is currently limited by several issues. Comparisons with the general population are limited because the CarerQol instrument questions are specifically targeted at the burden associated with being a carer. In addition, because of the novelty of the CarerQol instrument, few comparable studies currently exist. Unfortunately, the total CarerQol-7D score is often not reported. Nonetheless, several comparisons can be made as follows:

1. A large study of 1244 adult carers in the Netherlands reported a mean CarerQol-7D score of 79.1 and a mean CarerQol-VAS of 7.1 [26]. The mean CarerQol-7D of 64.1 and CarerQol-VAS of 6.3 detected in this study suggests higher carer burden and lower happiness in DCM carers compared with carers in general.
2. Similarly, CarerQol-VAS scores indicated that mean happiness in carers of individuals with DCM was lower than those observed in carers of individuals suffering from other conditions. Reported scores were 7.5 in head and neck cancers [31], 7.4 in autism [29], 7.5 in craniofacial malformations [32], and 7.2 in Pompe disease [13]. Therefore, DCM may pose heavier burden on carers than other reported diseases.
3. Using a similar 0 to 10 scale as in the CarerQol-VAS, the average population happiness has been estimated at 7.1 for the United Kingdom, 7.3 for the United States, 7.6 for the Netherlands, and 7.5 for Ireland [33]. The reduction of happiness in carers of individuals with DCM is large compared with the average happiness of the study population country (12.5%) and compared with carers of people with Pompe disease (5.3%), craniofacial malformations (2.7%), and autism (1.4%).

Although these comparisons are not without limitation, including lack of formal statistical comparison, carer quality of life scores appear consistently lower in DCM than reported in other diseases and general population estimates, suggesting that quality of life may be particularly affected in DCM carers. Exactly why carers are more affected in DCM remains unclear.

In this study we did not identify any associated factors. This may have been masked by our sample size, as studies involving larger groups (eg, n=200), albeit using alternative quality of life instruments, have found that carer demographics such as age, gender, health status, and duration of caregiving influence carer quality of life [34]. However, the absence of any trends between our investigated factors and carer quality of life may suggest that instead, significant factors were not considered. For example, other studies have shown a link with increasing hours of required care and the patient’s quality of life [13].

Recent research indicates that patient quality of life in DCM is lower than many other severe chronic conditions, including diabetes, cancers, and COPD [6]. In addition, DCM patients have a high prevalence of affective disorders [35], which may provide a possible explanation for the high carer burden and warrants further investigation.

### Generalizability

As CarerQol tariffs become available for several other countries [25], more nationalities should be included in future work. The comparison of diverse cultures and health care systems may provide novel insight into factors influencing carer quality of life. For example, it would be interesting to compare carer quality of life across cultures that place differing care expectations upon the family of those who are ill and between countries with more and less developed health and social care systems. This would help elucidate whether it is personal or wider societal factors that are most influential on carer quality of life.

### Limitations and Future Directions

Internet recruitment to patient-specific health surveys can be effective and reach an international audience efficiently and inexpensively, with less missing data compared with postal questionnaires [36,37].

Despite promising and comparable completion rates, the number of carers recruited was lower than patients in a DCM patient study using the same recruitment methods. Given the levels of disability in DCM, having fewer carers than patients is unlikely. It is more likely the internet recruitment methods were more effective for patients than carers, perhaps with patients more likely to identify with their condition (DCM) than carers with their situation (caring for someone with DCM). Given the limited experience of internet recruitment and behavior among carers, we can only speculate at this time. However, to support further research in this field, this deserves further consideration.

Inclusion of only 56 of the 136 responses received in the final analysis was disappointing. In total, 64% (56/87) of all responses from carers were included. A large proportion of excluded responses were inadvertently completed by patients, which is understandable given that at present most Myelopathy.org website visitors are patients. Nonetheless, finding no substantial differences between complete and incomplete responses makes selection bias unlikely. Moreover, the exclusion of carers from countries for which CarerQol tariffs were not available will resolve as more tariffs become available. Plans for development of a designated carer section of Myelopathy.org may improve carer participation in future work.

The participation rate of 32% in this study was lower than the typical response rate of 50% to 70% in other CarerQol studies [29,31,32]. These studies utilized postal questionnaires rather than open electronic questionnaires, as utilized in this study. Although response and participation rates are not identical measures, the participation rate in this study is almost certainly a substantial underestimate because it was not possible to distinguish between patient and carer website visitors, meaning that a large number of patient visitors were included in the denominator of our participation rate calculation.

Finally, patient and carer factors considered in this study were not found to influence carer quality of life. Although it is possible any associations may have been concealed by the sample size, considering other factors including the number of hours spent caring per week and further assessing the emotional and psychological burden for patient and carer will form important future work [13,35,38]. The identification of such factors is important to target supportive interventions.

### Conclusions

DCM carer quality of life is low. The magnitude of reduction in DCM carer quality of life appears greater than reductions in carer quality of life in other conditions studied. In this study, no single patient or carer factors were associated with carer quality of life. Identification of influencing factors is important to better understand the basis for impaired quality of life and to target support, which should form the basis of future work.

## Appendix

Multimedia Appendix 1 Kendall tau-b correlations between carer burden and happiness and patient disease severity and pain scores.

Multimedia Appendix 2 No significant difference in carer happiness was identified between the different groups of 9 demographic characteristics using 1-way analysis of variances.

## Abbreviations

ANOVA: analysis of variance
CarerQol: Care-Related Quality of Life
COPD: chronic obstructive pulmonary disease
DCM: degenerative cervical myelopathy
NIHR: National Institute for Health Research
VAS: Visual Analog Scale
