# Supplementary material for: Quality of Life Among Informal Caregivers of Patients With Degenerative Cervical Myelopathy: Cross-Sectional Questionnaire Study
Source: Interact J Med Res. 2019 Nov 7;8(4):e12381. doi: 10.2196/12381 (PMC6914271; doi:10.2196/12381)
Supplement: Multimedia Appendix 1 [file ijmr_v8i4e12381_app1.pdf]

Supplementary Table 1. Kendall's tau-b correlations between carer burden and happiness and patient disease severity and pain scores.

	**Nurick score**		**Current Neck Pain**		**Best Neck Pain**		**Worst Neck Pain**		**Current Arm/hand pain**		**Best Arm/Hand Pain**		**Worst Arm/Hand Pain**	
	τ_b	***p***	τ_b	***p***	τ_b	***p***	τ_b	***p***	τ_b	***p***	τ_b	***p***	τ_b	***p***
CarerQol-7D	-.20	.054	-.05	.582	-.09	.373	-.08	.446	-.10	.287	-.09	.368	-.10	.300
CarerQol-VAS	-.09	.408	.11	.270	.087	.398	.16	.128	-.01	.868	.09	.396	.04	.715
