# Supplementary material for: Quality of Life Among Informal Caregivers of Patients With Degenerative Cervical Myelopathy: Cross-Sectional Questionnaire Study
Source: Interact J Med Res. 2019 Nov 7;8(4):e12381. doi: 10.2196/12381 (PMC6914271; doi:10.2196/12381)
Supplement: Multimedia Appendix 2 [file ijmr_v8i4e12381_app2.pdf]

Supplementary Table 2. No significant difference in carer happiness was identified between the different groups of 9 demographic characteristics using one-way ANOVAs

**Factor**	**F**	**df1**	**df2**	**p**	**n**
Patient Age	1.21	4	51	.317	56
Patient Gender	.28	1	54	.597	56
Patient Education	1.00	4	51	.415	56
Patient Dependency	.00	1	54	.972	56
Carer Age	2.39	4	49	.064	54
Carer Gender	.04	1	51	.838	53
Carer Education	.48	5	47	.787	53
Carer Employment	.17	4	48	.954	53
Time as Carer	.81	3	12.5	.509	56
